# Reflections and Readiness of Medical Students for Self-Directed Learning (SDL): An Observational Cross-Sectional Study

**DOI:** 10.7759/cureus.82358

**Published:** 2025-04-16

**Authors:** Avula Naveen, A K Hairunnisa, Ashok K Dubey

**Affiliations:** 1 Pharmacology and Therapeutics, All India Institute Medical Sciences, Bilaspur, IND; 2 Internal Medicine, Andaman and Nicobar Islands Institute of Medical Sciences, Port Blair, IND; 3 Pharmacology and Therapeutics, All India Institute of Medical Sciences, Bilaspur, IND

**Keywords:** cognitive skills, medical education, readiness, reflections, self-directed learning

## Abstract

Background/introduction

Self-directed learning (SDL) is primarily an active learning technique that promotes higher-order cognitive skills and increases the self-efficacy of students. Through SDL, the onus of learning primarily lies with the students. Medical students need to be lifelong learners, as it is crucial to update their knowledge and apply the same in clinical practice. SDL plays a vital role in inculcating the habit of reading and learning in medical graduates. It also helps in developing all the domains of learning, such as cognitive, psychomotor, and affective.

Materials and methods

In this observational, questionnaire-based, cross-sectional study, a total of 208 first- and third-year medical students (104 students in each batch) were asked to fill out a pre-validated questionnaire form to record the reflections and readiness of the students regarding SDL in medical education. The participants were asked to select from a Likert scale of "strongly disagree," "disagree," "neutral," "agree," and "strongly agree." An open-ended questionnaire was used to record the views and reflections of students about the concept of SDL in medical education. Results were tabulated on an Excel sheet (Microsoft Corporation, Redmond, Washington), and descriptive statistical measures such as means and percentages were used for analysis of the data.

Results

Approximately 90 (86.53%) of third-year professional students responded positively to SDL readiness, whereas it was approximately 80 (76.92%) among first-year professional students. The majority of students, 198 (95.19%), revealed that the biggest challenges for the successful implementation of active learning through SDL are time constraints and the need for extensive planning and research skills.

Conclusions

Our study concluded that, overall, the third-year professional students showed higher reported readiness for learning the concepts through SDL in all three domains, such as motivation for learning, planning and implementation, and interpersonal communication skills.

## Introduction

Adult education expert Malcolm Knowles defines self-directed learning (SDL) as the process by which the students themselves take the initiative to diagnose their learning needs, formulate their learning goals, identify resources for learning, and evaluate their learning outcomes [1,2].

SDL is an active learning approach that enhances higher-order cognitive abilities and boosts students' self-confidence. In SDL, the responsibility for learning primarily falls on the students themselves [3-5]. For medical students, being lifelong learners is essential to stay updated with the latest knowledge and apply it effectively in clinical practice. SDL is instrumental in fostering the habit of continuous reading and learning among medical graduates, and it contributes to the development of all learning domains, including cognitive, psychomotor, and affective skills [6].

In Indian medical education, the new competency-based medical education (CBME) curriculum introduced several new concepts and strategies to make the learning process more interesting and learner-centric. CBME emphasizes student-centric active learning rather than teacher-centric passive learning. The Graduate Medical Education Regulations (GMR) 2019 stipulate learning with an emphasis on knowledge and skill acquisition through different strategies such as learning pedagogy, self-directed learning, flipped classroom, community-based learning, peer-assisted learning, use of online resources (e-learning), group learning, assessment-driven learning, simulation-based learning, problem-based learning, and learning from patients and other members of the healthcare team [7,8].

There has been a significant shift in how learning and information are accessed from various online platforms within the medical field. Medical students are increasingly turning to social media, podcasts, blogs, and similar resources for educational purposes. One notable example is FOAM (Free Open Access Meducation), which provides free, advanced, and innovative learning materials designed to help physicians and medical students refresh their knowledge and enhance their understanding in an engaging, motivating, and efficient manner [9,10].

While the benefits of being self-directed and the traits of a self-directed learner are well recognized, there is still ongoing debate about whether self-direction can be taught or applied in every context and across all educational levels in higher education. SDL is often viewed as a spectrum, present to varying degrees in individuals, with those who are least self-directed depending entirely on the instructor for their learning [11,12].

Despite the promises of active learning and progress in the field of medical education, the system of medical education in India has been skeptical about adapting to this change in pedagogy. SDL is an educational concept that has been receiving increasing attention since the implementation of CBME by the Medical Council of India (MCI) and the National Medical Council (NMC) [13,14].

There are very few studies regarding the implementation of SDL, students' readiness, and their reflections in the Indian context. This study intends to provide more insights into these areas of learning in medical education.

Medical teachers have a key role in helping students understand and develop the habit of adopting new strategies in active learning, especially SDL, that would ultimately guide them to achieve not only academic objectives but also life goals in the future.

Indian medical graduates differ from foreign medical graduates in terms of age, school education, learning patterns, entrance exams such as the National Eligibility cum Entrance Test (NEET), selection into medical courses, and family dependence. It is very important to assess the readiness and perception of medical students toward SDL in the curriculum. Our study evaluated the reflections of students on SDL so far in medical school. SDL is an evolving learning modality in medical education that Indian medical students should adopt, and teachers must train the students to gain the maximum benefits from SDL. Very few studies have been conducted regarding SDL readiness and reflections among Indian medical graduates, and this is the first of its kind among the students of the Andaman and Nicobar Islands.

## Materials and methods

 Study design and ethical considerations

The study was an observational, cross-sectional study. It was initiated after receiving approval from the Institutional Ethics Committee, Andaman and Nicobar Islands Institute of Medical Sciences (ANIIMS), no: ANIIMS/IEC/2022-23/27. Written informed consent was obtained after explaining the project details to each participant, and the confidentiality and anonymity of the participants were upheld throughout the study. The study was conducted in accordance with the guidelines set by the Institutional Ethics Committee.

Study participants and sampling

The study participants were the first- and third-year medical students of ANIIMS, Port Blair, the only medical college in the Andaman and Nicobar Islands of India. A convenience sample of 228 students (114 students in each batch of first- and third-year MBBS) was selected for the study. The study participants were requested to complete a pre-validated, structured questionnaire. The questionnaire was validated by three independent experts in medical education. The questionnaires were circulated among the students through Google Forms, and the responses were recorded in Google Sheets. Of the 228 students, only 208 provided complete and satisfactory responses to all the questions. Therefore, we used 208 responses as the sample size for the study; the remaining participants' data were excluded.

Data collection and statistical analysis

The questionnaire included various questions assessing students' readiness for and perceptions of SDL in medical education. The first section gathered demographic information such as age, gender, and year of study in medical school. The subsequent section focused on evaluating the medical students' readiness for SDL in their education. A self-made, pre-validated questionnaire was used to record the reflections and readiness of the students [15]. The questionnaire consisted of 20 questions, with the first six items focusing on students' learning motivation, questions 7 to 16 assessing their planning and implementation skills, and the remaining items addressing their interpersonal communication abilities. The participants were requested to choose from a Likert scale of "strongly disagree," "disagree," "neutral," "agree," and "strongly agree." Another questionnaire was used to record the views and reflections of students about the concept of SDL in medical education. The results were organized in an Excel sheet (Microsoft Corporation, Redmond, Washington), and statistical measures, including means and percentages, were applied as needed for analysis.

## Results

A total of 208 medical students participated in the study, comprising 104 students each from the first- and third-year professional MBBS batches. Among the participants, 112 (53.84%) were female students, and the remaining 96 (46.15%) were male students. The majority of participants belonged to the age group of 18 to 20 years. Table 1 illustrates the demographic details of the participants.

**Table 1:** Demographic profile of the study participants

Variables	Number (N)	Percentage (%)
Gender distribution of students		
Female	112	53.84%
Male	96	46.15%
Age distribution of students		
18-20 years	194	93.26%
21-23 years	11	5.28%
24-26 years	03	1.44%
Number of students from each professional year		
First professional year	104	50%
Third professional year	104	50%

The study also evaluated the comparison of readiness levels between first- and third-year professional medical students. It first assessed the quality of motivation for learning by asking different questions, as specified in Tables 2, 3. From question numbers 1 to 6, around 80% of first-year professional students showed positive responses toward motivation for learning the topic through SDL, and the percentage was slightly higher (90%) among third-year professional students.

In this category of motivation for learning through SDL, a maximum of 93 (89.42%) first-year professional students and 97 (93.26%) third-year professional students responded positively to question number 4, stating that they would be inspired by both successes and failures. The study revealed that third-year professional students were more prepared in terms of motivation for learning than the first-year professional students.

Regarding the concept of student readiness in the planning and implementation of SDL, based on questions 7 to 16, around 75% of first-year professional students and 85% of third-year professional students responded positively. Almost 91 (87.5%) first-year professional students indicated that they could connect new knowledge with their personal experiences and understand the strengths and weaknesses of their learning. When the same parameters were assessed in third-year professional students, approximately 99 (95.19%) responded positively.

The last part of the questionnaire, from questions 17 to 20, focused on the assessment of interpersonal communication skills. The study revealed that around 71% of first-year professional students responded positively to acquiring the necessary interpersonal communication skills for effective implementation of SDL in learning. The corresponding percentage was more than 80% among third-year professional students. The highest number of responses in this category was for question number 17, which indicated that interaction with others helps them plan for further learning, with 90 (86.53%) first-year professional students and 92 (88.46%) third-year professional students responding positively. Only 58 (55.76%) of first- and 76 (73.07%) of third-year professional students responded positively to question number 18, which indicated that they would like to learn the language and culture of those with whom they frequently interact.

Overall, the third-year professional students were better prepared for learning the concepts through SDL in all three domains: motivation for learning, planning and implementation, and interpersonal communication skills. Tables 2, 3 illustrate the results in first- and third-year professional students.

**Table 2:** Readiness of first-year professional students about self-directed learning in medical education

Question	Strongly Agree, N (%)	Agree, N (%)	Neutral, N (%)	Disagree, N (%)	Strongly Disagree, N (%)
1. I know what to learn.	18 (17.3)	56 (53.84)	26 (25)	3 (2.88)	1 (0.96)
2. I will continue to learn to improve my skills, irrespective of the results.	20 (19.23)	54 (51.92)	28 (26.92)	1 (0.96)	1 (0.96)
3. I hope I continue to excel in my learning.	45 (43.26)	47 (45.19)	9 (8.65)	2 (1.92)	1 (0.96)
4. I will be inspired by my successes and failures.	40 (38.46)	53 (50.96)	8 (7.69)	2 (1.92)	1 (0.96)
5. I enjoy finding answers to questions.	32 (30.76)	51 (49.03)	13 (12.5)	5 (4.8)	3 (2.88)
6. I will not give up learning if I face any difficulties.	36 (34.61)	50 (48.07)	12 (11.53)	4 (3.84)	2 (1.92)
7. I can proactively set my learning goals.	27 (25.96)	57 (54.8)	15 (14.42)	3 (2.88)	2 (1.92)
8. I know appropriate learning strategies in reaching my learning goals.	25 (24.03)	47 (45.19)	28 (26.92)	3 (2.88)	1 (0.96)
9. I can set my priorities of learning.	23 (22.11)	52 (50)	23 (22.11)	4 (3.84)	2 (1.92)
10. In the classroom or on my own, I can follow my plan of learning.	21 (20.19)	42 (40.38)	35 (33.65)	5 (4.8)	1 (0.96)
11. I am good at organizing my learning time.	24 (23.07)	41 (39.42)	33 (31.73)	4 (3.84)	2 (1.92)
12. I know how to find resources for my learning.	22 (21.15)	5 (53.84)	21 (20.19)	4 (3.84)	1 (0.96)
13. I can connect new knowledge with my personal experiences.	35 (33.65)	57 (54.8)	8 (7.69)	3 (2.88)	1 (0.96)
14. I can understand the strengths and weaknesses of my learning.	36 (34.61)	55 (52.88)	9 (8.65)	2 (1.92)	2 (1.92)
15. I can monitor my learning progress.	25 (24.03)	53 (50.96)	17 (16.34)	6 (5.76)	3 (2.88)
16. I can evaluate my learning outcomes.	28 (26.92)	57 (54.80)	16 (15.38)	2 (1.92)	1 (0.96)
17. My interactions with others aid me in planning better.	33 (31.73)	57 (54.80)	8 (7.69)	4 (3.84)	2 (1.92)
18. I would like to learn the language and culture of those with whom I frequently interact.	17 (16.34)	41 (39.42)	37 (35.57)	7 (6.73)	2 (1.92)
19. I can communicate my ideas clearly in oral presentations.	23 (22.11)	54 (51.92)	23 (22.11)	3 (2.88)	1 (0.96)
20. I can communicate messages effectively in writing.	21 (20.19)	52 (50)	25 (24.03)	5 (4.8)	1 (0.96)

**Table 3:** Readiness of third-year professional students about self-directed learning in medical education

Question	Strongly Agree, N (%)	Agree, N (%)	Neutral, N (%)	Disagree, N (%)	Strongly Disagree, N (%)
1. I know what to learn.	8 (26.92)	62 (59.61)	10 (9.61)	3 (2.88)	1 (0.96)
2. I will continue to learn to improve my skills, irrespective of the results.	32 (30.76)	58 (55.76)	11 (10.57)	2 (1.92)	1 (0.96)
3. I hope I continue to excel in my learning.	48 (46.15)	49 (47.11)	5 (4.8)	1 (0.96)	1 (0.96)
4. I will be inspired by my successes and failures.	44 (42.3)	53 (50.96)	4 (3.84)	2 (1.92)	1 (0.96)
5. I enjoy finding answers to questions.	36 (34.61)	58 (55.76)	8 (7.69)	1 (0.96)	1 (0.96)
6. I will not give up learning if I face any difficulties.	38 (36.53)	54 (51.92)	8 (7.69)	3 (2.88)	1 (0.96)
7. I can proactively set my learning goals.	34 (32.69)	58 (55.76)	9 (8.65)	2 (1.92)	1 (0.96)
8. I know appropriate learning strategies for reaching my learning goals.	33 (31.73)	56 (53.84)	11 (10.57)	3 (2.88)	1 (0.96)
9. I can set my priorities of learning.	31 (29.8)	54 (51.92)	14 (13.46)	4 (3.84)	1 (0.96)
10. In the classroom or on my own, I can follow my plan of learning.	28 (26.92)	49 (47.11)	21 (20.19)	5 (4.8)	1 (0.96)
11. I am good at organizing my learning time.	32 (30.76)	52 (50)	15 (14.42)	4 (3.84)	1 (0.96)
12. I know how to find resources for my learning.	27 (25.96)	59 (56.73)	13 (12.5)	4 (3.84)	1 (0.96)
13. I can connect new knowledge with my personal experiences	43 (41.34)	55 (52.88)	4 (3.84)	1 (0.96)	1 (0.96)
14. I can understand the strengths and weaknesses of my learning.	39 (37.5)	60 (57.69)	3 (2.88)	1 (0.96)	1 (0.96)
15. I can monitor my learning progress.	32 (30.76)	55 (52.88)	12 (11.53)	4 (3.84)	1 (0.96)
16. I can evaluate my learning outcomes.	36 (34.61)	57 (54.8)	8 (7.69)	2 (1.92)	1 (0.96)
17. My interactions with others aid me in planning better.	34 (32.69)	58 (55.76)	9 (8.65)	2 (1.92)	1 (0.96)
18. I would like to learn the language and culture of those with whom I frequently interact.	25 (24.03)	51 (49.03)	24 (23.07)	3 (2.88)	1 (0.96)
19. I can communicate my ideas clearly in oral presentations.	28 (26.92)	59 (56.73)	13 (12.5)	3 (2.88)	1 (0.96)
20. I can communicate messages effectively in writing.	26 (25)	58 (55.76)	18 (17.3)	1 (0.96)	1 (0.96)

Assessment of challenges and reflections

Around 198 (95.19%) students were of the opinion that the biggest challenge for the implementation of active learning through SDL was that it demands a lot of effort and time from both faculty and students, and it also requires extensive planning and research skills. Next, around 196 (94.23%) students believed that the vastness of the syllabus and the limited time frame for the course were barriers to the effective implementation of SDL in the medical curriculum. More than 178 (85.57%) students reflected that distractions due to modern technology, difficulty in assessing student performance, and challenges in understanding complex subjects were obstacles they encountered in the SDL learning strategy. More than 136 (65.38%) students thought that further training of teachers is required for the optimal execution of SDL in medical education. Only around 72 (43.61%) students identified difficulty in accessing technology as a major barrier to the effective implementation of SDL. Table 4 illustrates the results on challenges.

**Table 4:** Challenges for self-directed learning adaptation among medical students

S. No.	Question	Yes, N (%)	No, N (%)	I Don’t Know, N (%)
1	Vast syllabus, limited time frame	196 (94.23)	8 (3.84)	4 (1.92)
2	Distractions due to modern technology	178 (85.57)	27 (12.98)	3 (1.44)
3	Difficulty in accepting a novel method	189 (90.86)	13 (6.25)	6 (2.88)
4	Complex subjects cannot be fully taught	172 (82.69)	31 (14.9)	5 (2.4)
5	It demands a lot of effort and time from both faculty and students	198 (95.19)	7 (3.36)	3 (1.44)
6	It needs too much planning and research skills	198 (95.19)	7 (3.36)	3 (1.44)
7	Problems with team participation	148 (71.15)	58 (27.88)	2 (0.96)
8	Poor training of the teachers in handling the sessions	136 (65.38)	62 (29.80)	10 (4.8)
9	Assessment of performance is difficult	176 (84.61)	28 (13.46)	4 (1.92)
10	Difficulty in accessing technology	72 (43.61)	136 (65.38)	0 (0)

Advantages of SDL in medical education

More than 192 (92.3%) students agreed that SDL provides motivation to learn new concepts, offers a joyful learning experience, allows flexibility in time and space to learn, and also develops critical thinking in learners. Only around 136 (65.38%) students responded positively about SDL's role in promoting teamwork and time management. Table 5 illustrates these findings.

**Table 5:** Advantages of self-directed learning in medical education

S. No.	Question	Yes, N (%)	No, N (%)	I Don’t Know, N (%)
1	It gives motivation to learn new concepts	192 (92.3)	12 (5.76)	4 (1.92)
2	Improving time management	138 (66.34)	64 (30.76)	8 (3.84)
3	It enables knowledge application	182 (87.5)	21 (10.09)	5 (2.4)
4	It helps identify skills and self-assessment	172 (82.69)	29 (13.94)	7 (3.36)
5	It develops critical thinking in learners	196 (94.23)	8 (3.84)	4 (1.92)
6	It promotes teamwork	136 (65.38)	66 (31.73)	6 (2.88)
7	It provides a joyful experience for learning	198 (95.19)	6 (2.88)	4 (1.92)
8	It gives flexibility in time and space to learn	196 (94.23)	8 (3.84)	4 (1.92)

## Discussion

In our study, all the students shared similar educational and cultural backgrounds, and they were all exposed to SDL to the same degree. Studies have revealed that students with poor academic performance may find it difficult to adopt and cope with SDL, whereas students with good academic performance tend to do well in SDL. This disparity could be due to a lack of abilities such as information gathering and processing, analytic skills, and communication skills among low achievers [16,17].

The study revealed that around 86% of third-year professional students responded positively to SDL readiness, whereas it was around 76% in the case of first-year professional students. In the students' educational background, insufficient exposure to SDL may explain why first-year students reported a lower level of self-directedness compared to those in their third professional year. Our findings align with a previous study that showed students in the problem-based learning (PBL) curriculum became progressively better at self-directed learning as they advanced through their years of study [18]. However, our results differ from a cross-sectional study on a hybrid curriculum at Dalhousie University Faculty of Medicine in Canada, which found no difference in SDL scores between first- and second-year students [19]. Our findings also contrast with those of cross-sectional studies from the University of Toronto Faculty of Medicine and Indonesian medical schools, where first-year students had significantly higher SDL scores than second-year students, possibly due to the initial enthusiasm among first-year students at the start of the curriculum [20,21]. Furthermore, other longitudinal studies tracking SDL readiness in students enrolled in PBL hybrid and traditional medical curricula have shown a notable decline in SDL readiness as students progress to higher stages of medical training [22]. For the successful implementation of SDL, a medical teacher plays a vital role as a facilitator: introducing the topic, furnishing authentic information to minimize the students' effort, maintaining learning standards, and teaching students about self-evaluation. Trained teachers are key to the effective implementation of SDL. Even in our study, more than 65.38% of students believed that further training of teachers is required for the optimal execution of SDL in medical education, which aligns with findings from other studies [23,24].

In the latest CBME curriculum in India, it is mandatory to implement SDL. The National Medical Council (NMC) has even specified a fixed number of hours for each subject. Though it was introduced in 2019, there are still some challenges that need to be addressed for the effective implementation of SDL. Both teachers and students should move out of their comfort zones and adopt newer teaching-learning strategies for joyful and effective learning. For SDL to be successfully implemented in medical education, both government and private institutions should work in tandem to prepare faculty with adequate skills and provide them with sufficient resources and technological support. Teachers must constantly motivate students to adopt and practice SDL in their formative years to stay updated with new developments in medical education.

Limitations of the study

The study was conducted among medical graduate students from the Andaman and Nicobar Islands Institute of Medical Sciences, the only medical college in the entire Islands, and the sample used in the study was limited to two batches. Therefore, the results obtained may not be applicable to medical students from other parts of India. Participants might also have responded in favor of widely accepted statements rather than expressing their personal views or beliefs. The inclusion of medical graduates from additional batches and the integration of SDL readiness with assessment results could have provided a deeper understanding of how SDL influences students' academic performance. The questionnaire was distributed only in English and administered through online Google Forms, which might have excluded students with language difficulties, poor internet access, or limited digital skills. Therefore, further large-scale studies involving a more diverse student population are needed to gain a clearer understanding of medical students' perceptions of SDL and its implementation.

## Conclusions

Our study concluded that third-year professional students reported higher readiness to learn concepts through SDL in all three domains: motivation for learning, planning and implementation, and interpersonal communication skills. The majority of students indicated that the biggest challenge to the successful implementation of active learning through SDL was time constraints, as it requires extensive planning and research skills. Most participants agreed that SDL provides motivation to learn new concepts, offers a joyful learning experience, allows flexibility in time and space to learn, and also develops critical thinking in learners. Curriculum with flexible learning modules, the use of technology, integration of assessment methods with SDL, faculty training, promotion of critical thinking, and continuous feedback from students are important steps for the effective implementation of the CBME curriculum in India.

## References

[1] Shah DK, Yadav RL, Sharma D, Yadav PK, Sapkota NK, Jha RK, Islam MN (2016). Learning approach among health sciences students in a medical college in Nepal: a cross-sectional study. Adv Med Educ Pract.

[2] Greveson GC, Spencer JA (2005). Self-directed learning--the importance of concepts and contexts. Med Educ.

[3] Khiat H (2017). Academic performance and the practice of self-directed learning: the adult student perspective. J Furth High Educ.

[4] Kidane HH, Roebertsen H, van der Vleuten CP (2020). Students' perceptions towards self-directed learning in Ethiopian medical schools with new innovative curriculum: a mixed-method study. BMC Med Educ.

[5] Ananth Krishnan N (2018). Competency-based undergraduate curriculum for the Indian Medical Graduate, the new MCI curricular document: positives and areas of concern. J Basic Clin Appl Health Sci.

[6] Cadorin L, Bressan V, Palese A (2017). Instruments evaluating the self-directed learning abilities among nursing students and nurses: a systematic review of psychometric properties. BMC Med Educ.

[7] Tang F, Chen C, Zhu Y (2017). Comparison between flipped classroom and lecture-based classroom in ophthalmology clerkship. Med Educ Online.

[8] Arya V, Gehlawat VK, Rana R, Kaushik J (2020). Flipped classroom versus traditional lecture in training undergraduates in pediatric epilepsy. J Family Med Prim Care.

[9] Young TP, Bailey CJ, Guptill M, Thorp AW, Thomas TL (2014). The flipped classroom: a modality for mixed asynchronous and synchronous learning in a residency program. West J Emerg Med.

[10] Premkumar K, Pahwa P, Banerjee A, Baptiste K, Bhatt H, Lim HJ (2013). Does medical training promote or deter self-directed learning? A longitudinal mixed-methods study. Acad Med.

[11] El-Gilany AH, Abusaad Fel S (2013). Self-directed learning readiness and learning styles among Saudi undergraduate nursing students. Nurse Educ Today.

[12] Fisher MJ, King J (2010). The self-directed learning readiness scale for nursing education revisited: a confirmatory factor analysis. Nurse Educ Today.

[13] Rajalakshmi M, Ganapathy K (2023). Effect of self-directed learning module and assessment on learning of national health programme by medical undergraduates - a mixed methods evaluation. Indian J Community Med.

[14] Rajashree R, Chandrashekar DM (2020). Competency-based medical education in India: a work in progress. Indian J Physiol Pharmacol.

[15] Shen WQ, Chen HL, Hu Y (2014). The validity and reliability of the self-directed learning instrument (SDLI) in mainland Chinese nursing students. BMC Med Educ.

[16] Levett-Jones TL (2005). Self-directed learning: implications and limitations for undergraduate nursing education. Nurse Educ Today.

[17] Bhat PB, Rajashekar B, Kamath U (2007). Perspectives on self-directed learning—the importance of attitudes and skills. Biosci Educ.

[18] Schmidt HG, Vermeulen L, van der Molen HT (2006). Longterm effects of problem-based learning: a comparison of competencies acquired by graduates of a problem-based and a conventional medical school. Med Educ.

[19] Lee YM, Mann KV, Frank BW (2010). What drives students' self-directed learning in a hybrid PBL curriculum. Adv Health Sci Educ Theory Pract.

[20] Leatemia LD, Susilo AP, van Berkel H (2016). Self-directed learning readiness of Asian students: students perspective on a hybrid problem based learning curriculum. Int J Med Educ.

[21] Harvey BJ, Rothman AI, Frecker RC (2003). Effect of an undergraduate medical curriculum on students' self-directed learning. Acad Med.

[22] Premkumar K, Vinod E, Sathishkumar S, Pulimood AB, Umaefulam V, Prasanna Samuel P, John TA (2018). Self-directed learning readiness of Indian medical students: a mixed method study. BMC Med Educ.

[23] Dolmans DH, De Grave W, Wolfhagen IH, van der Vleuten CP (2005). Problem-based learning: future challenges for educational practice and research. Med Educ.

[24] Wood DF (2003). Problem based learning. BMJ.

